# Association between chronic kidney disease and oxidative balance score: National Health and Nutrition Examination Survey (NHANES) 2005–2018

**DOI:** 10.3389/fnut.2024.1406780

**Published:** 2025-01-03

**Authors:** Cong Liu, Jiju Yang, Hongdian Li, Yuanyuan Deng, Pengfei He, Jiao Zhang, Mianzhi Zhang

**Affiliations:** ^1^Department of Nephrology, Dongfang Hospital of Beijing University of Chinese Medicine, Beijing, China; ^2^Graduate School of Beijing University of Chinese Medicine, Beijing, China; ^3^Department of Nephrology, Tianjin Academy of Traditional Chinese Medicine Affiliated Hospital, Tianjin, China; ^4^Tianjin Famous Chinese Medicine Inheritance Workshop of Mianzhi Zhang, Tianjin, China

**Keywords:** chronic kidney disease, oxidative balance score, NHANES, a cross-sectional study, dietary components

## Abstract

**Background:**

The oxidative balance score (OBS) is a comprehensive concept that includes 16 dietary components and four lifestyle factors to assess an individual's exposure to pro-oxidants and antioxidants. This study aims to explore the relationship between OBS and the risk of chronic kidney disease (CKD).

**Methods:**

This cross-sectional study included nationally representative National Health and Nutrition Examination Survey (NHANES) participants aged 18 and above from 2005 to 2018. The OBS, a novel concept derived from multiple dietary (pro-oxidant and antioxidant nutrients) and lifestyle exposures (including smoking, alcohol consumption, obesity, and physical activity), serves as a useful tool for assessing an individual's oxidative stress status. The continuous variable OBS was converted into categorical variables by quartiles. Covariates included age, gender, race, education level, marital status, poverty-income ratio, sleep duration, depression, hypertension, diabetes, hyperlipidemia, cardiovascular disease, use of hypoglycemic medications, and use of antihypertensive medications. The relationship between OBS and CKD was explored using multiple logistic regression analysis and restricted cubic spline models. Additionally, subgroup analyses, interaction tests, and sensitivity analyses were conducted to validate the stability of the results.

**Results:**

A total of 25,118 NHANES participants were included in this study. The weighted prevalence of CKD was 14.97%. In the fully adjusted model, compared to the lowest OBS quartile, participants in the highest quartile had a 26% reduced risk of CKD (OR = 0.74, 95%CI: 0.63–0.87, *p* < 0.001). In restricted cubic spline regression, there was a linear association between OBS and CKD. The results of subgroup analysis and sensitivity analysis remain consistent. A significant interaction was found in the stratified analysis by age group (*p* for interaction = 0.012), suggesting that individuals older than 60 years may benefit more significantly from an increase in OBS scores compared to those aged 60 years or younger.

**Conclusion:**

This study demonstrates that higher OBS is associated with a lower risk of CKD, particularly among the elderly population, providing innovative insights and preliminary evidence for the development of preventive strategies against CKD.

## 1 Introduction

Chronic kidney disease (CKD) has emerged as a significant public health issue globally, posing a threat to human health. Both early-stage CKD and complications of renal failure are highly prevalent, consuming substantial medical resources (1, 2). According to the latest Global Burden of Disease report (3), the global median prevalence of CKD was 9.5%, with the highest prevalence observed in Eastern and Central Europe. The annual median costs associated with kidney replacement therapy were: $19,380 per person for hemodialysis, $18,959 for peritoneal dialysis, and $26,903 for the first year of kidney transplantation. The escalating prevalence rates and the substantial socioeconomic burden imposed by end-stage renal disease underscore the urgency for physicians to actively adopt preventive strategies in clinical practice. However, there remains a lack of evidence-based lifestyle recommendations for primary prevention of CKD, emphasizing the need for innovative research on modifiable risk factors and preventive strategies.

Oxidative stress refers to the process of cellular oxidative damage induced by the accumulation of reactive oxygen species (ROS), which is a crucial factor in inducing renal fibrosis (4, 5). Elevated levels of oxidative stress are already present in the early stages of CKD (6, 7). As the disease progresses, the levels of pro-oxidant biomarkers gradually increase, while those of antioxidant biomarkers, such as glutathione peroxidase and carbonyl stress biomarkers, decrease (8, 9). However, measuring these biomarkers requires specialized techniques and is challenging to implement, thus limiting their clinical application. The high interconnectivity of the biological molecular network in the body also poses limitations, as it fails to comprehensively consider the intricate and interdependent interactions among biomolecules (10). Additionally, oxidative stress levels are influenced by various factors, including diet and lifestyle habits, rendering individual biomarkers insufficient to accurately reflect an individual's oxidative stress status. Thus, the oxidative balance score (OBS) (11) has been developed to evaluate an individual's exposure to pro-oxidants and antioxidants, with a higher score indicating greater antioxidant activity.

Previous studies have found that OBS is associated with a variety of diseases, including chronic obstructive pulmonary disease (12), kidney stones (13), and cardiovascular disease (CVD) (14), depression (15). A cohort study has shown that a higher OBS is associated with a lower risk of CKD development among the Korean population (16). However, two studies (17, 18) conducted in the US population have yielded inconsistent results, with Haibin Wen et al. suggesting a linear negative correlation between OBS and CKD without a clear inflection point, while Yuewei Yin et al. proposing a nonlinear negative relationship between the two. To further clarify the association between OBS and CKD and explore whether this correlation differs among populations with distinct characteristics, the present study aims to conduct a cross-sectional analysis using data from the National Health and Nutrition Examination Survey (NHANES), with the goal of providing new evidence-based insights into the potential of OBS as an innovative strategy for CKD prevention.

## 2 Materials and methods

### 2.1 Study population

This study is a nationwide cross-sectional research that draws data from the NHANES conducted during the 2005–2018 survey cycles. NHANES is administered by the National Center for Health Statistics, which employs a stratified multistage probability sampling technique to conduct household interviews, physical examinations, and laboratory tests among participants, aiming to assess the health and nutritional status of the non-institutionalized civilian population in the US. NHANES has received approval from the National Center for Health Statistics Ethics Review Committee, and all participants have provided written informed consent.

Participants in this study underwent screening based on the following criteria, individuals were excluded if (1) < 18 years; (2) incomplete data of CKD; (3) items comprising the OBS were < 16; (4) incomplete data of covariates.

### 2.2 Creatinine and albumin measurements and CKD

Serum and urinary creatinine were measured by the Jaffe rate method, and urinary albumin was measured by a solid-phase fluorescent immunoassay. For estimated glomerular filtration rate (eGFR) calculation, we applied the CKD-Epidemiology Collaboration (19) as follows: GFR = 141·min [Scr/κ,1]α × max [Scr/κ, 1] – 1.209 × 0.993 Age × 1.018 [if women] × 1.159 [if black]; κ was 0.7 (women) or 0.9 (men), a was −0.329 (women) or −0.411 (men), and min/max indicate the minimum/maximum of Scr/κ or 1. CKD (20) was diagnosed based on the low eGFR (< 60 mL/min/1.73 m^2^) or urinary albumin-to-creatinine ratio (ACR) of more than 30 mg/g.

### 2.3 OBS

OBS is calculated based on 16 dietary nutrients and four lifestyle factors. The dietary nutrients include: dietary fiber, carotene, riboflavin, niacin, vitamin B6, total folic acid, vitamin B12, vitamin C, vitamin E, calcium, magnesium, zinc, copper, selenium, total fat, and iron. The intake of these nutrients is obtained through 24-h dietary recall interviews, and the intake of each nutrient is calculated using the University of Texas Food Intake Analysis System and the US Department of Agriculture's Survey Nutrient Database. The four lifestyle factors include physical activity, body mass index (BMI), alcohol consumption, and smoking status, with smoking intensity represented by serum cotinine levels. Physical activity is calculated based on the total metabolic equivalent of task level of various exercises performed within a week (metabolic equivalent of each physical activity × frequency of physical activity × duration). Obesity scores are assigned based on weight status: obesity [BMI ≥ 30 kg per square meter (kg/m^2^)], overweight (25 ≤ BMI < 30 kg/m^2^), and normal weight (BMI < 25 kg/m^2^). Among these, pro-oxidant factors include total fat, total iron intake, smoking status, alcohol consumption, and obesity status, while the rest are antioxidant factors. The calculation of the OBS total score is as follows (Supplementary Table 1): alcohol consumption is categorized into three groups based on daily intake: non-drinkers, moderate drinkers (females 0–15 g/d, males 0–30 g/d), and heavy drinkers (females ≥ 15 g/d, males ≥ 30 g/d), with scores of 2, 1, and 0 respectively. Obesity, overweight, and normal weight are assigned scores of 0, 1, and 2 respectively. Other components are grouped into tertiles based on gender, with antioxidant factors in tertiles 1–3 assigned scores of 0–2, and pro-oxidant factors in tertiles 1–3 assigned scores of 2–0. The OBS total score is obtained by summing up the scores of all components, with a higher OBS total score indicating a higher level of antioxidant exposure.

### 2.4 Assessment of covariates

Based on previous studies (21–23), and in conjunction with our research objectives, potential covariates included age, gender, marital status (divorced/separated/widowed, married/living with a partner, never married), race/ethnicity (non-Hispanic White, non-Hispanic Black, Mexican American and other), education level (less than high school, high school diploma, more than high school), the family poverty income ratio [PIR(< 1.3, 1.3-3.5, >3.5)], sleep duration, depressive status (as assessed by The Patient Health Questionnaire), hypertension (taking antihypertensive medications, diagnosed by a doctor, systolic blood pressure ≥ 140 mmHg or diastolic blood pressure ≥90 mmHg) (24), diabetes mellitus (using diabetes medications or insulin, diagnosed by a doctor, glycated hemoglobin HbA1c ≥6.5%, fasting glucose ≥7 mmol/L, random blood glucose ≥11.1 mmol/L, 2-h oral glucose tolerance test with blood glucose ≥11.1 mmol/L) (25), hyperlipidemia (taking cholesterol-lowering drugs, total cholesterol ≥200 mg/dL, triglyceride ≥150 mg/dL, low-density lipoprotein ≥130 mg/dL, or high-density lipoprotein ≤ 50 mg/dL for women and ≤ 40 mg/dL for men), CVD(coronary heart disease, congestive heart failure, heart attack, stroke, angina), use of hypoglycemic medications, and use of antihypertensive medications.

### 2.5 Statistical analyses

This study utilized R software (version 4.3.3) for all statistical analyses. Given the stratified and complex multi-stage sampling design of NHANES, we weighted the data according to the sample weighting method recommended by NHANES, combining data from 2005 to 2018, with the 14-year weight equal to 1/7 of the 2-year weight. Continuous variables are presented as mean ± sd, while categorical variables are represented as weighted proportions. A Shapiro-Wilk statistical test was employed to confirm the normal distribution of continuous variables. Variables with skewed distributions are presented as medians and quartiles. We categorized the continuous variable OBS into quartiles and used Weighted Analysis of Variance or Chi-square tests to assess differences between OBS (quantile) groups. Weighted Logistic regression models were employed to analyze the association between OBS and CKD, with results reported as odds ratios (OR) with their 95% confidence intervals (95% CI) and *p*-values, and trend *p*-values were calculated. Three models were used in this study: Model 1 was adjusted for sociodemographic characteristics including gender, age, marital status, race, education level, and family PIR; Model 2 further adjusted for comorbidities in Model 1: diabetes, hyperlipidemia, hypertension, CVD, and depression; Model 3 adjusted for all covariates. In addition to conducting subgroup analyses to explore the specific relationships between OBS and CKD in different gender, age, and status of diabetes, hypertension, hyperlipidemia, and CVD, we also performed interaction tests to determine whether the effect of OBS on CKD risk was consistent across populations with different characteristics, i.e., whether there were significant interactions. A multivariate-adjusted restricted cubic spline model with three knots was constructed to establish an OR curve, exploring the presence of a nonlinear dose-response association between OBS and CKD. Furthermore, to validate the stability of the results, after employing multiple imputation with five replications and the chained equation to handle missing data, the logistic regression results were compared between the imputed dataset and the dataset with original values. A two-sided *p* < 0.05 was considered statistically significant.

## 3 Results

### 3.1 Baseline characteristics

**Figure 1:**
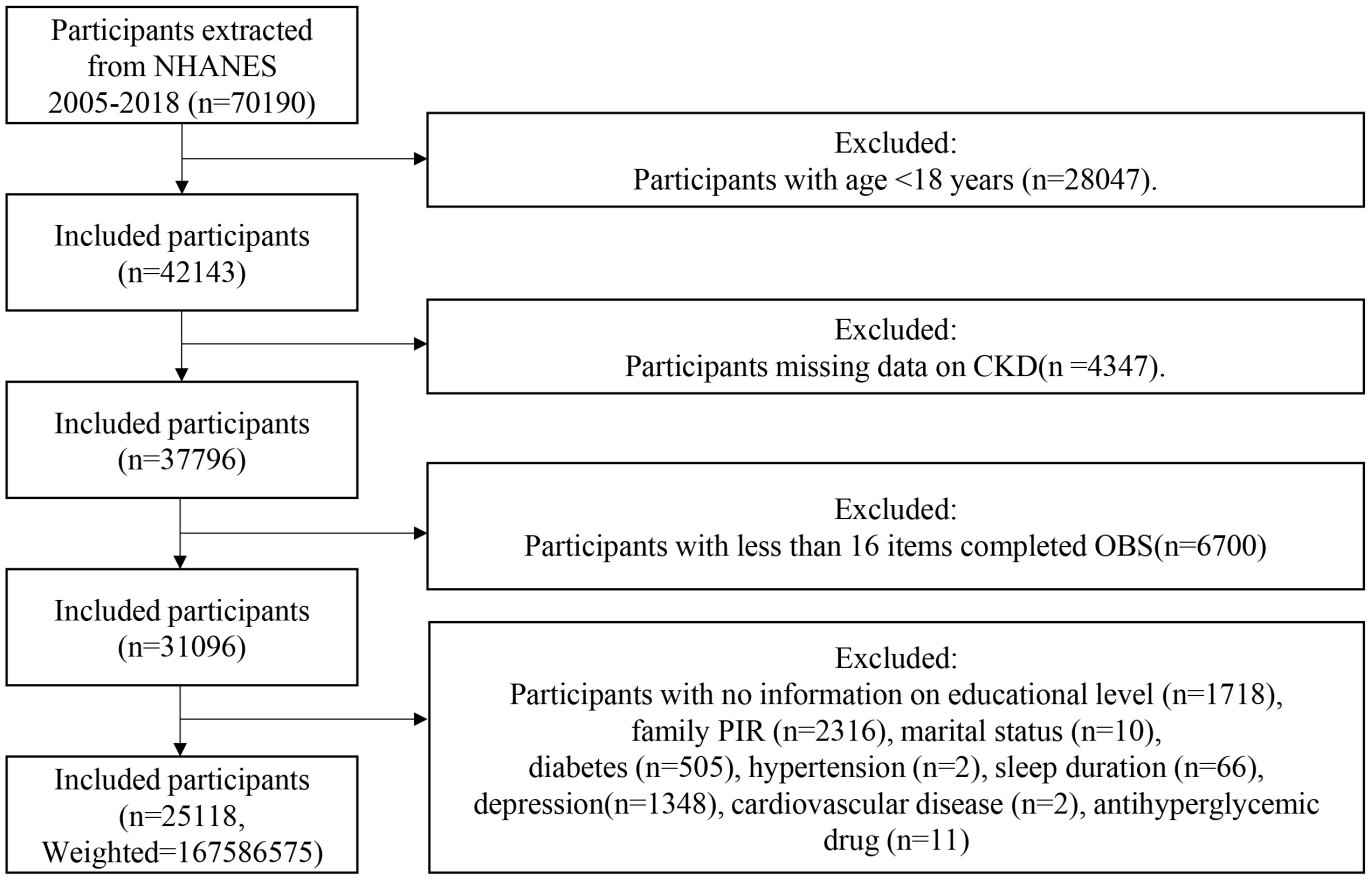
Flowchart of the sample selection from NHANES 2005–2018.

After subject screening (as shown in Figure 1), 25,118 NHANES participants were included in the study, representing 167.6 million non-institutionalized residents of the US. Table 1 presents the baseline characteristics of the study participants grouped according to OBS quartiles. The mean age of the participants was 48.29 ± 16.78 years, with 51.67% being female, and the weighted prevalence of CKD was 14.97%. Compared to the participants in the lowest OBS quartile, those in the highest OBS quartile were younger, had longer sleep duration, higher levels of education, were more likely to be married or have a partner, had higher wealth levels, and had a higher proportion of Non-Hispanic White individuals. The prevalence of hypertension, diabetes, hyperlipidemia, CVD, depression, and CKD also decreased with increasing OBS. There was no statistically significant difference in gender distribution between the different OBS groups.

**Table 1:** Characteristics of participants by quartiles of the OBS in the NHANES 2005–2018 cycles.

**Variables**	**Total**	**Q1, [3,15]**	**Q2, [15,20]**	**Q3, [20,26]**	**Q4, [26,37]**	***p* value**
	***n*** = **167,586,575**	***n*** = **39,523,166**	***n*** = **35,366,578**	***n*** = **48,197,204**	***n*** = **44,499,627**	
Age, years	48.29 ± 16.78	49.10 ± 17.43	48.75 ± 17.18	48.08 ± 16.54	47.43 ± 16.05	0.0029
**Sex, %**						0.5069
Male	48.33	49.11	48.43	47.31	48.68	
Female	51.67	50.89	51.57	52.69	51.32	
**Race and ethnicity, %**						< 0.0001
Non-Hispanic White	70.46	64.17	69.55	71.84	75.28	
Non-Hispanic Black	10.17	16.75	10.97	8.39	5.62	
Mexican American	7.66	7.42	8.11	8.05	7.08	
Other	11.71	11.66	11.36	11.72	12.01	
**Educational level, %**						< 0.0001
Less than high school	13.86	21.60	15.40	11.72	8.05	
High school diploma	23.47	29.75	26.82	22.05	16.76	
More than high school	62.68	48.65	57.78	66.23	75.19	
**Marriage status, %**						< 0.0001
Divorced/separated/widowed	18.85	23.29	20.18	17.37	15.45	
Married/living with a partner	63.79	57.67	63.17	65.80	67.56	
Never married	17.36	19.04	16.66	16.83	16.99	
**Family PIR, %**						< 0.0001
< 1.3	20.30	29.95	21.20	17.53	14.00	
1.3–3.5	35.24	39.47	36.72	34.84	30.75	
≥3.5	44.46	30.58	42.08	47.63	55.25	
**Hypertension, %**						< 0.0001
Yes	38.85	44.51	41.46	38.11	32.55	
No	61.15	55.49	58.54	61.89	67.45	
**Hyperlipidemia, %**						< 0.0001
Yes	71.72	75.04	74.36	72.41	65.94	
No	28.28	24.96	25.64	27.59	34.06	
**Diabetes mellitus, %**						< 0.0001
Yes	14.27	18.65	15.94	13.90	9.47	
No	85.73	81.35	84.06	86.10	90.53	
**Cardiovascular disease, %**						< 0.0001
Yes	8.96	13.12	10.08	7.67	5.76	
No	91.04	86.88	89.92	92.33	94.24	
Sleep duration, hours	7.13 ± 1.42	7.06 ± 1.67	7.11 ± 1.42	7.14 ± 1.36	7.19 ± 1.24	0.0056
**Depression, %**						< 0.0001
Yes	76.98	69.05	76.19	78.52	82.98	
No	23.02	30.95	23.81	21.48	17.02	
Yes	8.82	11.42	10.01	9.00	5.37	
No	91.18	88.58	89.99	91.00	94.63	
**Antihypertensive drug, %**						< 0.0001
Yes	28.56	33.27	31.24	27.92	22.95	
No	71.44	66.73	68.76	72.08	77.05	
ACR, mg/g	6.55 (4.32,11.77)	7.15 (4.58,14.44)	6.77 (4.36,12.23)	6.46 (4.32,11.06)	6.12 (4.14,10.63)	< 0.0001
eGFR, mL/min/1.73 m^2^	93.04 ± 21.76	91.91 ± 24.22	92.18 ± 22.47	93.72 ± 21.16	93.98 ± 19.31	0.0001
**CKD, %**						< 0.0001
Yes	14.97	19.69	16.47	13.50	11.19	
No	85.03	80.31	83.53	86.50	88.81	

OBS, oxidative balance score; Q, quartiles; NHANES: National Health and Nutrition Examination Survey; CKD, chronic kidney disease; PIR, poverty income ratio.

ACR, urinary albumin-to-creatinine ratio; eGFR, estimated glomerular filtration rate.

Data presented as weighted percentage for categorical variables and mean+ SD for continuous variables, and skewed distribution variables as medians and quartiles.

### 3.2 Association between OBS and CKD

As shown in Table 2, weighted logistic regression analysis revealed a negative association between OBS and CKD. In the fully adjusted model, compared to the lowest OBS quartile, participants in the highest quartile had a 26% reduced risk of CKD (OR = 0.74, 95%CI: 0.63–0.87, *p* < 0.001). The trend remained relatively stable across different models. Additionally, compared to the lowest quartile, participants in the highest quartile of dietary OBS had a 26% reduced risk of CKD (OR = 0.74, 95%CI: 0.63-0.88, *p* < 0.001), and those in the highest quartile of lifestyle OBS had a 19% reduced risk of CKD (OR = 0.81, 95%CI: 0.68-0.97, *p* = 0.021).

**Table 2:** Weighted logistic regression analysis models showing the associations between OBS and CKD.

**Variable**	**OBS levels quartile**							***p* for trend**
	**Q1**	**Q2**		**Q3**		**Q4**		
	**OR (95%CI)**	**OR (95%CI)**	***p*** **value**	**OR (95%CI)**	***p*** **value**	**OR (95%CI)**	***p*** **value**	
**OBS**								
Unadjusted	1.00(ref)	0.80 (0.71–0.91)	< 0.001	0.64 (0.57–0.71)	< 0.001	0.51 (0.45–0.59)	< 0.001	< 0.001
Model 1	1.00(ref)	0.87 (0.76–1.00)	0.05	0.75 (0.66–0.84)	< 0.001	0.65 (0.56–0.76)	< 0.001	< 0.001
Model 2	1.00(ref)	0.89 (0.77–1.02)	0.103	0.78 (0.69–0.88)	< 0.001	0.74 (0.63–0.87)	< 0.001	< 0.001
Model 3	1.00(ref)	0.88 (0.77–1.02)	0.083	0.77 (0.68–0.87)	< 0.001	0.74 (0.63–0.87)	< 0.001	< 0.001
**Dietary OBS**								
Unadjusted	1.00(ref)	0.82 (0.71–0.94)	0.004	0.63 (0.57–0.70)	< 0.001	0.57 (0.49–0.65)	< 0.001	< 0.001
Model 1	1.00(ref)	0.87 (0.75–1.01)	0.059	0.75 (0.67–0.84)	< 0.001	0.70 (0.60–0.82)	< 0.001	< 0.001
Model 2	1.00(ref)	0.88 (0.76–1.01)	0.069	0.77 (0.68–0.86)	< 0.001	0.74 (0.63–0.88)	< 0.001	< 0.001
Model 3	1.00(ref)	0.87 (0.76–1.01)	0.062	0.76 (0.67–0.86)	< 0.001	0.74 (0.63–0.88)	< 0.001	< 0.001
**Lifestyle OBS**								
Unadjusted	1.00(ref)	0.68 (0.60–0.76)	< 0.001	0.60 (0.53–0.67)	< 0.001	0.51 (0.44–0.59)	< 0.001	< 0.001
Model 1	1.00(ref)	0.68 (0.60–0.78)	< 0.001	0.65 (0.57–0.75)	< 0.001	0.61 (0.51–0.72)	< 0.001	< 0.001
Model 2	1.00(ref)	0.76 (0.66–0.87)	< 0.001	0.77 (0.67–0.88)	< 0.001	0.80 (0.68–0.96)	0.014	< 0.001
Model 3	1.00(ref)	0.76 (0.66–0.88)	< 0.001	0.76 (0.67–0.87)	< 0.001	0.81 (0.68–0.97)	0.021	< 0.001

Model 1: Adjusted for age, sex, race, marriage status, education and family PIR.

Model 2: Adjusted for model 1 + diabetes mellitus, hypertension, hyperlipidemia, cardiovascular disease, depression.

Model 3: Adjusted for model 2 + sleep duration, antihyperglycemic drug, antihypertensive drug.

OBS, oxidative balance score; CKD, chronic kidney disease; Q, quartiles; OR, odds ratio; 95% CI, 95% confidence interval.

**Figure 2:**
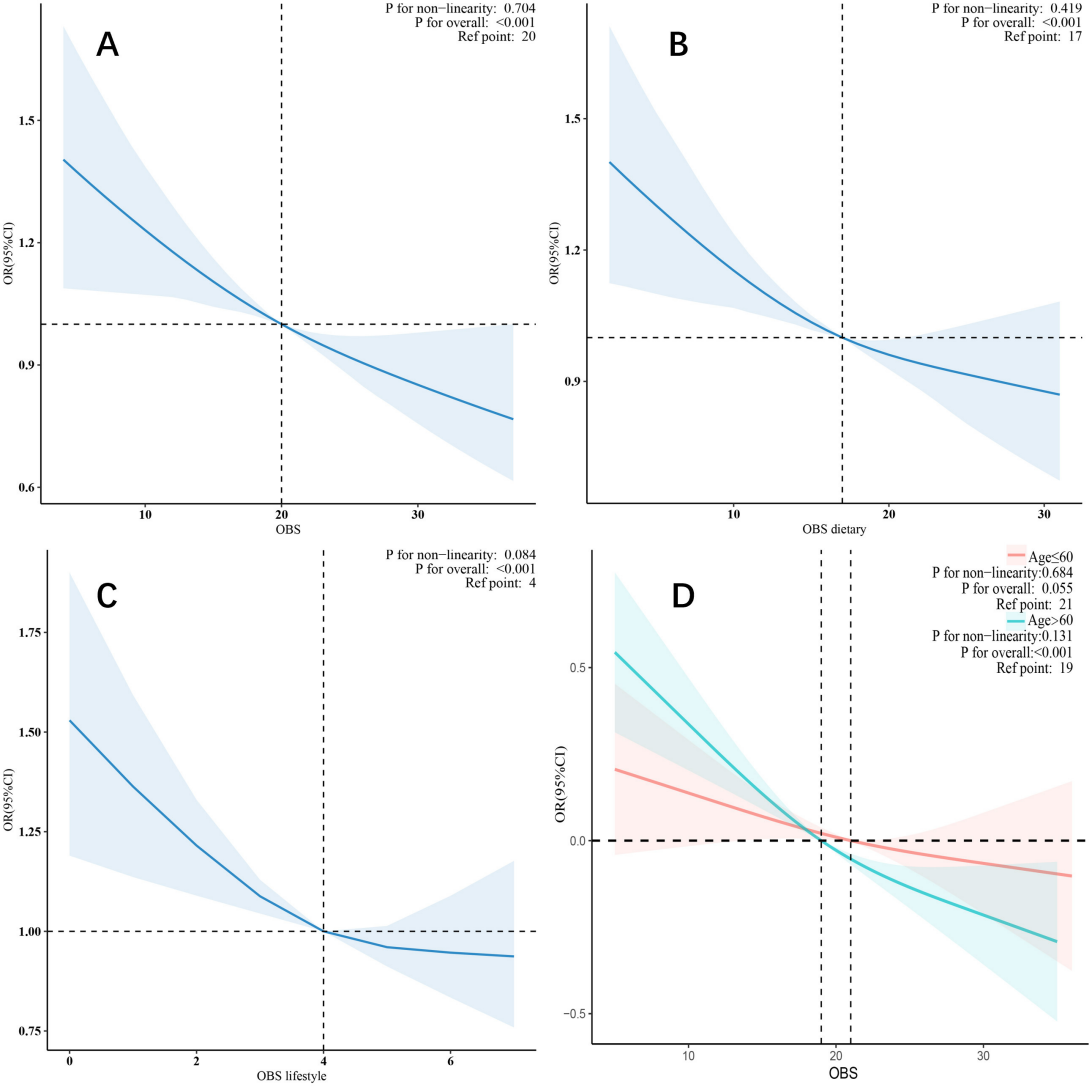
**(A–D)** Multivariable-adjusted restricted cubic spline model.

In the restricted cubic spline model, after adjusting for all covariates, a linear relationship was observed between OBS, dietary OBS, lifestyle OBS (Figures 2A–C), and CKD. As OBS increased, the risk of CKD gradually decreased.

### 3.3 Subgroup analyses and sensitivity analysis

**Figure 3:**
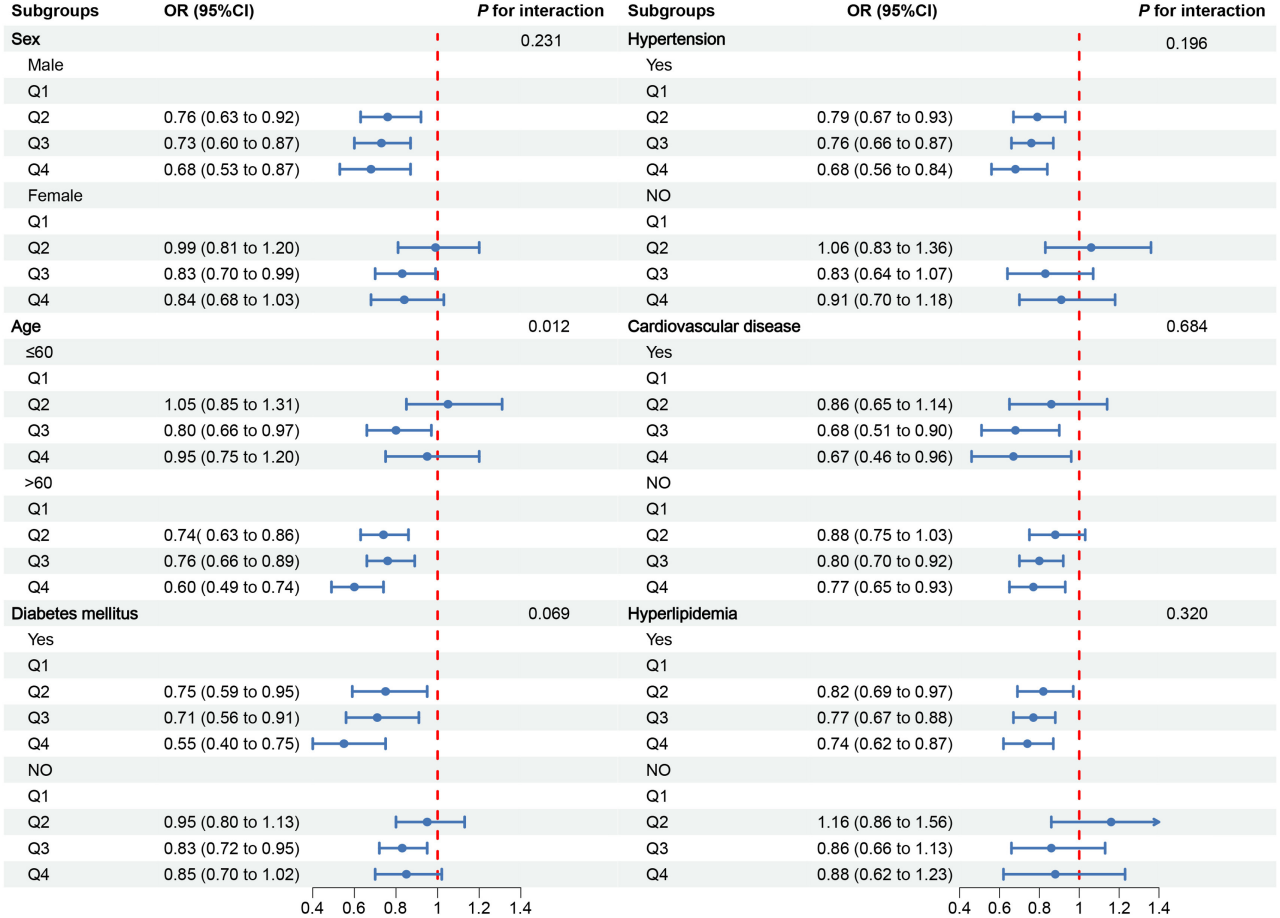
Associations between OBS and CKD in different subgroups.

To further explore the relationship between OBS and CKD, we conducted subgroup analyses in different population contexts and tested for interactions (Figure 3). In the CVD subgroups, a negative correlation was observed between OBS and CKD. However, in other subgroups, the negative correlation was only observed in males, individuals older than 60 years, those with diabetes, hypertension, and hyperlipidemia. Notably, a significant interaction was found in the stratified analysis by age group (*p* for interaction = 0.012), suggesting that individuals older than 60 years may benefit more significantly from an increase in OBS compared to those aged 60 years or younger (Figure 2D).

To further validate the robustness of our findings, we applied multiple imputation methods to generate five imputed datasets. Multivariate logistic regression analyses were conducted on each of the five imputed datasets, and similar results were obtained in the fully adjusted models, confirming the negative association between OBS and the risk of CKD (Supplementary Table 2).

## 4 Discussion

We conducted a cross-sectional analysis involving 25,118 participants from the NHANES database. Our findings revealed a negative association between OBS and the risk of CKD, with this relationship remaining consistent across subgroup and sensitivity analyses. Furthermore, we observed a more pronounced correlation between OBS and CKD in the subgroup of participants aged over 60 years. Collectively, our study provides preliminary evidence for the exploration of the correlation between OBS and CKD, offering novel insights for future clinical and basic research endeavors.

OBS is an index that integrates 20 factors to assess pro-oxidants and antioxidants exposure. The kidney is rich in mitochondria and is highly susceptible to oxidative stress (26). Studies (9, 27, 28) have shown that patients with CKD have elevated levels of pro-oxidant biomarkers and significantly lower levels of antioxidant biomarkers. As a major source of bioactive components, diet mediates redox reactions and regulates the body's oxidative homeostatic system (29). Study (30) has reported that low molecular weight antioxidants, such as vitamin C, vitamin E, and carotenoids, have protective effects against chronic diseases, these antioxidants activate the Nrf2 antioxidant factor. Additionally, a large cohort study based on a UK sample bank showed that increased fiber intake is associated with a reduced risk of CKD. A systematic review (31) has suggested that diets rich in dietary fiber may slow the progression of CKD and its associated complications, and advocated the development of dietary plans high in dietary fiber for CKD patients. Additionally, a high-level intake of carotene may reduce the risk of death in CKD populations due to its antioxidant properties. Vitamin C, also known as ascorbic acid, is a crucial water-soluble antioxidant. Elevated serum levels of vitamin C have been shown to reduce the risk of albuminuria, low eGFR, and CKD (32). Appropriately increasing serum vitamin C levels may help protect renal function, especially in the elderly. Additionally, vitamin C may improve oxidative stress in patients with type 2 diabetes mellitus by modulating the immune response (33). Folic acid, betaine, vitamins B6 and B12 can also delay the progression of diabetes mellitus and protect the kidney by degrading homocysteine through methylation (34).

Exogenous oxidative balance is influenced by various lifestyle factors, including smoking, alcohol consumption, and physical activity in OBS (35). A meta-analysis (36) of 103 observational studies found that a diet high in potassium and low in sodium, as well as vegetables, moderate alcohol consumption, higher levels of physical activity, and cessation of smoking, all of which are components of OBS, can reduce the risk of CKD. Research (37) has demonstrated that physical activity can decrease the risk of kidney damage and rapid decline in kidney function through mechanisms such as up-regulating endothelial nitric oxide and other antioxidant enzymes (38, 39). Additionally, adopting a healthy diet and lifestyle can reduce the risk of obesity and overweight (40), which are also significant factors in the development of CKD (41). Furthermore, smoking is strongly linked to an increased risk of CKD and proteinuria. This is due to its potential to cause insulin resistance, advanced glycosylation end-products, and increased renal vascular permeability. Smoking is a risk factor for kidney damage especially in diabetic patients. Notably, multiple studies (42–44) have shown that moderate alcohol consumption is associated with a lower risk of CKD compared to no alcohol consumption or heavy drinking. However, considering the differences in the classification of alcohol intake in the world, as well as the potential risk of alcohol consumption to CVD, it is still not recommended for non-drinkers to start drinking, and the appropriate alcohol intake of the general population still needs further research to determine that once excessive alcohol consumption will lead to the production of a large amount of ROS, reduce the antioxidant activity of glutathione peroxidase (45) and cause damage to the kidneys and heart, drinking alcohol is still a risk factor that needs attention.

The subgroup analysis revealed a consistent negative correlation between OBS and CKD within the CVD subgroups. However, in other subgroups, this negative correlation was only significantly observed among males, individuals older than 60 years, and those with diabetes, hypertension, or hyperlipidemia. Notably, age-stratified analysis uncovered a significant interaction, which may be attributed to the increased accumulation of ROS and decreased antioxidant capacity with advancing age (46). Gender differences may stem from hormonal influences, as male androgen testosterone potentially exacerbates oxidative stress (47). Abnormalities in glucose metabolism, elevated blood pressure, and dyslipidemia can all diminish antioxidant enzyme activity and oxidative stress clearance capacity (48, 49). Despite controlling for covariates to minimize interference during analysis, the limited sample size remains a potential limitation, potentially contributing to the randomness and uncertainty of the results. Therefore, these conclusions should be treated with caution, and further research is warranted to validate these preliminary findings.

This study boasts several strengths. Firstly, assessing OBS through dietary and lifestyle questionnaires provides an economical and efficient means to evaluate the balance between exogenous pro-oxidants and antioxidants. Our findings of a significant correlation between OBS and CKD, particularly in individuals over 60, pave the way for future longitudinal studies to explore the mechanisms underlying OBS's impact on CKD and ascertain age as a crucial factor. Once these results are further confirmed and expanded, they may inform or refine public health policies, such as emphasizing specific antioxidant-rich diets and lifestyles in the health management of elderly populations. Secondly, NHANES's utilization of a multistage complex probability sampling method enhances the generalizability of our findings within the US context.

However, this study also acknowledges several limitations. Firstly, antioxidants exhibit a threshold effect, where excess levels may exhibit pro-oxidant properties. Yet, OBS is defined under the assumption of a linear relationship between all components and oxidative stress, and its assessment via questionnaires is prone to recall and selection biases in reporting nutrient intake. Secondly, as this study was conducted in the US population, the racial universality of our findings remains to be further validated. Thirdly, despite constructing multivariate logistic regression models and conducting subgroup and sensitivity analyses to mitigate confounding factors, residual confounding effects cannot be entirely ruled out. Lastly, as a cross-sectional study, this research cannot establish a causal relationship between OBS and CKD, highlighting the need for more prospective studies in this area.

## 5 Conclusions

This study demonstrates that higher OBS is associated with a lower risk of CKD, particularly among the elderly. This suggests that OBS has the potential to serve as an important reference in clinical practice for structuring dietary patterns and lifestyles, offering innovative insights and preliminary evidence for developing preventive strategies against CKD. Nevertheless, further large-scale prospective studies are warranted to validate and expand upon our findings.

## Data Availability

The datasets presented in this study can be found in online repositories. The names of the repository/repositories and accession number(s) can be found below: https://www.cdc.gov/nchs/nhanes/index.htm.
