# Supplementary material for: Association between chronic kidney disease and oxidative balance score: National Health and Nutrition Examination Survey (NHANES) 2005–2018
Source: Front Nutr. 2025 Jan 3;11:1406780. doi: 10.3389/fnut.2024.1406780 (PMC11738952; doi:10.3389/fnut.2024.1406780)
Supplement: Supplementary file 1 [file Table_1.docx]

Supplementary Table 1 Components of the oxidative balance score.

| OBS components | Property | Male | | | Female | | |
| --- | --- | --- | --- | --- | --- | --- | --- |
|  |  | 0 | 1 | 2 | 0 | 1 | 2 |
| Dietary OBS components | | | | | | | |
| Dietary fiber (g/d) | A | ≤13.1 | 13.1-20.65 | ＞20.65 | ≤11.1 | 11.1-16.95 | ＞16.95 |
| Carotene (RE/d) | A | ≤52.104 | 52.104-175.75 | ＞175.75 | ≤55.375 | 55.375-193.229 | ＞193.229 |
| Riboflavin (mg/d) | A | ≤1.744 | 1.744-2.556 | ＞2.556 | ≤1.37 | 1.37-1.969 | ＞1.969 |
| Niacin (mg/d) | A | ≤22.266 | 22.266-32.056 | ＞32.056 | ≤16.08 | 16.08-22.909 | ＞22.909 |
| Vitamin B_6_ (mg/d) | A | ≤1.71 | 1.71-2.551 | ＞2.551 | ≤1.278 | 1.278-1.878 | ＞1.878 |
| Total folate (mcg/d) | A | ≤324.5 | 324.5-491.5 | ＞491.5 | ≤256.5 | 256.5-379.5 | ＞379.5 |
| Vitamin B_12_ (mcg/d) | A | ≤3.595 | 3.595-6.19 | ＞6.19 | ≤2.575 | 2.575-4.47 | ＞4.47 |
| Vitamin C (mg/d) | A | ≤41.55 | 41.55-100.75 | ＞100.75 | ≤39.9 | 39.9-89.7 | ＞89.7 |
| Vitamin E (ATE) (mg/d) | A | ≤5.935 | 5.935-9.44 | ＞9.44 | ≤4.935 | 4.935-7.81 | ＞7.81 |
| Calcium (mg/d) | A | ≤734 | 734-1120.5 | ＞1120.5 | ≤609.5 | 609.5-917.5 | ＞917.5 |
| Magnesium (mg/d) | A | ≤255.5 | 255.5-359.5 | ＞359.5 | ≤205 | 205-285.5 | ＞285.5 |
| Zinc (mg/d) | A | ≤9.705 | 9.705-14.23 | ＞14.23 | ≤7.13 | 7.13-10.29 | ＞10.29 |
| Copper (mg/d) | A | ≤1.04 | 1.04-1.483 | ＞1.483 | ≤0.85 | 0.85-1.202 | ＞1.202 |
| Selenium (mcg/d) | A | ≤100.9 | 100.9-142.2 | ＞142.2 | ≤73.5 | 73.5-104.5 | ＞104.5 |
| Total fat (g/d) | P | ＞100.67 | 68.05-100.67 | ≤68.05 | ＞75.25 | 51.24-75.25 | ≤51.24 |
| Iron (mg/d) | P | ＞18.395 | 12.53-18.395 | ≤12.53 | ＞13.99 | 9.68-13.99 | ≤9.68 |
| Lifestyle OBS components | | | | |  |  |  |
| Physical activity (MET-minute/week) | A | ≤1200 | 1200-4800 | ＞4800 | ≤720 | 720-2520 | ＞2520 |
| Alcohol (g/d) | P | ≥30 | 0-30 | None | ≥15 | 0-15 | None |
| Body mass index (kg/m^2^) | P | ≥30 | 25-30 | ＜25 | ≥30 | 25-30 | ＜25 |
| Cotinine (ng/mL) | P | ＞1.05 | 0.023-1.05 | ≤0.023 | ＞1.103 | 0.016-1.103 | ≤0.016 |

OBS: oxidative balance score; A: antioxidant; P: prooxidant; RE: retinol equivalent; ATE: alpha-tocopherol equivalent; MET: metabolic equivalent.
