# Supplementary material for: Association between chronic kidney disease and oxidative balance score: National Health and Nutrition Examination Survey (NHANES) 2005–2018
Source: Front Nutr. 2025 Jan 3;11:1406780. doi: 10.3389/fnut.2024.1406780 (PMC11738952; doi:10.3389/fnut.2024.1406780)
Supplement: Supplementary file 2 [file Table_2.docx]

Supplementary Table 2： Sensitivity analysis

| Variable | OBS levels quartile | | | | | | | *p* for trend |
| --- | --- | --- | --- | --- | --- | --- | --- | --- |
|  | Q1 | Q2 |  | Q3 |  | Q4 |  |  |
|  | OR (95%CI) | OR (95%CI) | *p* value | OR (95%CI) | *p* value | OR (95%CI) | *p* value |  |
| Imputed dataset 1 |  |  |  |  |  |  |  |  |
| Unadjusted | 1.00 (ref) | 0.81 (0.72-0.90) | <0.001 | 0.64 (0.57-0.70) | <0.001 | 0.51 (0.45-0.58) | <0.001 | <0.001 |
| Model 1 | 1.00 (ref) | 0.87 (0.77-0.98) | 0.020 | 0.74 (0.67-0.83) | <0.001 | 0.64 (0.56-0.74) | <0.001 | <0.001 |
| Model 2 | 1.00 (ref) | 0.89 (0.78-1.00) | 0.057 | 0.78 (0.70-0.87) | <0.001 | 0.73 (0.63-0.85) | <0.001 | <0.001 |
| Model 3 | 1.00 (ref) | 0.88 (0.78-1.00) | 0.042 | 0.78 (0.70-0.87) | <0.001 | 0.73 (0.63-0.85) | <0.001 | <0.001 |
| Imputed dataset 2 |  |  |  |  |  |  |  |  |
| Unadjusted | 1.00 (ref) | 0.81 (0.72-0.90) | <0.001 | 0.64 (0.57-0.70) | <0.001 | 0.51 (0.45-0.58) | <0.001 | <0.001 |
| Model 1 | 1.00 (ref) | 0.87 (0.77-0.98) | 0.021 | 0.74 (0.67-0.83) | <0.001 | 0.64 (0.55-0.74) | <0.001 | <0.001 |
| Model 2 | 1.00 (ref) | 0.89 (0.78-1.00) | 0.059 | 0.78 (0.70-0.87) | <0.001 | 0.73 (0.63-0.85) | <0.001 | <0.001 |
| Model 3 | 1.00 (ref) | 0.88 (0.78-1.00) | 0.045 | 0.78 (0.70-0.87) | <0.001 | 0.73 (0.63-0.85) | <0.001 | <0.001 |
| Imputed dataset 3 |  |  |  |  |  |  |  |  |
| Unadjusted | 1.00 (ref) | 0.81 (0.72-0.90) | <0.001 | 0.64 (0.57-0.70) | <0.001 | 0.51 (0.45-0.58) | <0.001 | <0.001 |
| Model 1 | 1.00 (ref) | 0.87 (0.77-0.98) | 0.020 | 0.74 (0.67-0.83) | <0.001 | 0.64 (0.55-0.74) | <0.001 | <0.001 |
| Model 2 | 1.00 (ref) | 0.89 (0.78-1.00) | 0.056 | 0.78 (0.70-0.87) | <0.001 | 0.73 (0.63-0.85) | <0.001 | <0.001 |
| Model 3 | 1.00 (ref) | 0.88 (0.78-1.00) | 0.043 | 0.78 (0.70-0.87) | <0.001 | 0.73 (0.63-0.85) | <0.001 | <0.001 |
| Imputed dataset 4 |  |  |  |  |  |  |  |  |
| Unadjusted | 1.00 (ref) | 0.81 (0.72-0.90) | <0.001 | 0.64 (0.57-0.70) | <0.001 | 0.51 (0.45-0.58) | <0.001 | <0.001 |
| Model 1 | 1.00 (ref) | 0.86 (0.77-0.98) | 0.020 | 0.74 (0.66-0.82) | <0.001 | 0.64 (0.55-0.74) | <0.001 | <0.001 |
| Model 2 | 1.00 (ref) | 0.89 (0.78-1.00) | 0.055 | 0.78 (0.70-0.87) | <0.001 | 0.73 (0.63-0.84) | <0.001 | <0.001 |
| Model 3 | 1.00 (ref) | 0.88 (0.78-1.00) | 0.042 | 0.78 (0.70-0.87) | <0.001 | 0.73 (0.63-0.85) | <0.001 | <0.001 |
| Imputed dataset 5 |  |  |  |  |  |  |  |  |
| Unadjusted | 1.00 (ref) | 0.81 (0.72-0.90) | <0.001 | 0.64 (0.57-0.70) | <0.001 | 0.51 (0.45-0.58) | <0.001 | <0.001 |
| Model 1 | 1.00 (ref) | 0.87 (0.77-0.98) | 0.020 | 0.74 (0.67-0.83) | <0.001 | 0.64 (0.56-0.74) | <0.001 | <0.001 |
| Model 2 | 1.00 (ref) | 0.89 (0.78-1.00) | 0.057 | 0.78 (0.70-0.87) | <0.001 | 0.73 (0.63-0.85) | <0.001 | <0.001 |
| Model 3 | 1.00 (ref) | 0.88 (0.78-1.00) | 0.043 | 0.78 (0.70-0.87) | <0.001 | 0.73 (0.63-0.85) | <0.001 | <0.001 |

Model 1: Adjusted for age, sex, race, marriage status, education and family PIR.

Model 2: Adjusted for model 1 + diabetes mellitus, hypertension, hyperlipidemia, cardiovascular disease and depression.

Model 3: Adjusted for model 2 + hypoglycemic medications, antihypertensive medications and sleep time.
